# Core-Shell Imprinted Particles for Adenovirus Binding

**DOI:** 10.3390/ma14247692

**Published:** 2021-12-13

**Authors:** Sandra Dietl, Paul Walther, Harald Sobek, Boris Mizaikoff

**Affiliations:** 1 Institute of Analytical and Bioanalytical Chemistry, Ulm University, Albert-Einstein-Allee 11, 89081 Ulm, Germany; sandra.dietl@uni-ulm.de; 2 Zentrale Einrichtung Elektronenmikroskopie, Ulm University, Albert-Einstein-Allee 11, 89081 Ulm, Germany; paul.walther@uni-ulm.de; 3 Labor Dr. Merk & Kollegen GmbH, Beim Braunland 1, 88416 Ochsenhausen, Germany; hs-ir@t-online.de

**Keywords:** virus imprinting, molecularly imprinted polymers, MIPs, adenovirus, core-shell imprinting, synthetic receptors

## Abstract

Virus-imprinted polymers were synthesized via surface imprinting strategies to produce core-shell imprinted particles selective for human adenovirus type 5. High binding affinity of the target virus towards the resulting imprinted layer was confirmed and unspecific binding was reduced in presence of blocking agents, i.e., via bovine serum albumin and skim milk in combination with Tween 20. In addition, the imprinted materials were applied for adenovirus extraction from cell culture supernatants. High levels of virus binding with negligible binding of matrix proteins confirmed the suitability of these materials for binding and extraction of the target virus from complex matrices.

## 1. Introduction

Molecular imprinted polymers are a promising alternative as sorbent materials for enrichment, solid-phase extraction or detection of different analyte species [1,2]. The use of a template to synthesize the imprinted layer results in high affinity and selectivity of the target analyte towards the recognition material. Hence, molecularly imprinted polymers (MIPs) are used for various targets, ranging from small organic molecules to larger biomacromolecular structures [3]. There is also the possibility to tailor the synthesis strategy according to the used target and respective application such that a variety of imprinted polymers can be fabricated including core-shell particles [4], magnetic materials [5], or thin films [6]. These MIPs have a high selectivity towards the target analyte with overall low matrix influences.

While MIPs for low molecular weight molecules are nowadays considered an established technology, imprinting of macromolecules like proteins or entire biological specimen such as viruses still faces some challenges, attributed to the nature of the target [4]. Large structures can adopt different conformations in solution, have low stability in organic solvents, and contain a variety of functional groups, which could result in increased unspecific binding [3]. However, imprinting of biomolecules gained higher awareness during the last years, since MIPs have several advantages over natural receptors, including higher stability, lower production costs, and better availability [7,8,9].

Attention towards the detection and enrichment of viruses using imprinted sorbents strongly increased during the last years [10]. Hence, virus-imprinted polymers (VIPs) for various viruses including tobacco mosaic virus [11], poliovirus [12], and influenza A viruses [13] are reported. Application of VIPs for virus detection can have various motivations, like analysis of waterborne viruses in the environment [14]. Other applications include virus detection in biological samples, like determination of tobacco mosaic virus in plant sap of tobacco leaves [15], or for medical purposes like the detection of hepatitis A in human serum samples [16].

The present study focused on the development of virus-imprinted polymers for the selective detection of human adenovirus type 5 (AdV5). For that purpose, core-shell particles were synthesized via surface imprinting strategies using silica particles as the core substrates. These particles offered an ideal surface chemistry for the subsequent functionalization and polymerization of an organic shell layer. The amino groups of lysine of the viral capsid were selected as anchor groups to form a covalent bond between template and aldehyde groups on the surface of the silica particles. The imine bond formation is reversible and can be cleaved under acidic conditions. High virus binding to the core particles was obtained and the polymer layer was formed by organosilane monomers. During rebinding of the template virus to the VIPs and the non-imprinted polymers (NIPs), the influence of protein blocking agents, including bovine serum albumin (BSA) and skim milk, on the nonspecific binding behavior of the virus was examined. In addition, the extraction of adenovirus from a complex matrix, i.e., cell culture supernatant (CCS) was investigated. In previous studies, the VIPs already showed high virus binding from CCS suspensions [17]. Therefore, binding of matrix proteins and nucleic acids was further characterized in this study to examine the application of the produced VIPs for adenovirus extraction from a complex matrix.

Adenoviruses are non-enveloped DNA-viruses with a protein capsid consisting of three viral proteins, i.e., the hexon, penton, and fiber proteins [18]. Various diseases are caused by adenoviruses, including respiratory, ocular, and gastrointestinal infections [19,20]. Adenoviruses are transmitted through different pathways, like aerosols or through water, for example in insufficiently disinfected swimming pools [21]. Imprinted particles could therefore be applied for adenovirus detection or enrichment in environmental samples, e.g., water samples, or for virus removal from aerosols. Additionally, virus infections could be detected in biological or medicinal samples. For this purpose, the investigation of adenovirus binding towards the synthesized imprinted particles from cell culture supernatants was one part of the herein reported study. In contrast, adenoviruses can be applied as viral vectors in gene therapy [22] such as the currently developed vector-based COVID-19 vaccines [23]. For this aim, imprinted particles might be used during virus purification steps as a fast, cheap, and scalable alternative compared to more expensive antibody-based affinity materials.

## 2. Materials and Methods

### 2.1. Materials

Tetraethylorthosilicate (TEOS), ethanol absolute, ammonia (28%), and glutaraldehyde (50% solution) were purchased from Merck (Darmstadt, Germany). The (3-Aminopropyl)triethoxysilane (APTES) 98% and (3-aminopropyl)trimethoxysilane (APTMS) 97% were purchased from Alfa Aesar (Kandel, Germany). Bovine serum albumin (BSA) ≥96% was purchased from SigmaAldrich (Taufkirchen, Germany). Quantitative polymerase chain reaction (qPCR) primer and probe were purchased from ThermoFisher (Darmstadt, Germany). CAPITAL^TM^ qPCR probe master mix was purchased from Biotechrabbit (Berlin, Germany). Mini-PROTEAN TGX gels, TRIS/glycine/SDS (TGS) running buffer, molecular weight marker precision plus protein dual Xtra prestained protein standard, and Laemmli sample preparation buffer were purchased from Bio-Rad (Feldkirchen, Germany). Quick Coomassie stain and Tween 20 were purchased from Serva Electrophoresis GmbH (Heidelberg, Germany). Difco^TM^ skim milk was purchased from BD Biosciences (Heidelberg, Germany). Phosphate-buffered saline (PBS) and cell-free cell culture supernatant from Chinese hamster ovary cells (CHO) in PBS were obtained from Labor Dr. Merk and Kollegen (Ochsenhausen, Germany). Human adenovirus type 5 (AdV5), initially purchased from ATCC VR-5, was propagated in A549 cells and purified at Labor Dr. Merk and Kollegen (Ochsenhausen, Germany). Viral DNA was purified using PureLink^TM^ Viral RNA/DNA mini kit (ThermoFisher). Viral lysis buffer contained in this kit was used for virus extraction. Viral DNA was purified according to the instructions of the manufacturer.

### 2.2. Synthesis of Functionalized Core Particles

Silica particles (SP) were synthesized according to the Stöber method [24]. First, 70 mL (1.2 mol) ethanol, 40 mL (1.8 mol) ammonia, and 20 mL water were mixed in a round bottom flask and stirred at 600 rpm. Then, 10 mL (0.045 mol) TEOS were added and the mixture was stirred for 20 h at 600 rpm and room temperature. After centrifugation, the particles were washed with ethanol and water and dried at 40 °C. Surface area of the dried silica particles was determined by nitrogen adsorption-desorption isotherms at 77.3 K on Quadrasorb-SI surface area analyzers (Quantachrome) and surface area was calculated using the Brunauer-Emmett-Teller (BET) method. For functionalization, silica particles (20 mg/mL) were suspended in water by ultrasonication, APTES (1.5 µmol/mg SP) was added, and the mixture was stirred at 500 rpm for 30 min. The particles were washed with water and resuspended, followed by the addition of glutaraldehyde (6 µmol/mg SP). After 30 min reaction time (500 rpm), the particles were washed with water, dried, and stored at RT until further use.

### 2.3. Monitoring of Functionalization

In order to monitor the functionalization process, samples were taken after each step for analysis by photoelectron spectroscopy (XPS) and zeta-potential. For zeta-potential determination, particle suspensions were diluted with 1 mM KCl, equilibrated at 25 °C, and analyzed with a Zetasizer Nano ZS (Malvern). XPS was performed with dried particles on a PHI 5800 (Physical Electronics). Amino-functionalized particles were additionally incubated with a ninhydrin solution (2% ninhydrin in ethanol) and heated to 95 °C for 10 min. Dried particles were also analyzed with a vario micro cube elemental analyzer (Elementar).

### 2.4. Scanning Electron Microscopy (SEM)

Particles were characterized after each step of the synthesis to verify uniform size distribution of the core particles and to monitor the imprinting procedure. Particle suspensions of bare silica particles and functionalized silica particles were applied on a silicon chip and after drying, the samples were used for imaging on a Quanta FEG 3D (FEI) without further pretreatment. Particles from imprinting and rebinding suspensions were prefixed in a fixation medium containing 5% glutaraldehyde for safety reasons due to their contact with virus. The particles were washed, fixed on a silicon chip, dried, and coated with platinum. For verification of virus binding, VIP samples were additionally treated with osmium tetroxide, fixed on a silicon chip, dried, and coated with carbon. Samples were imaged on a Hitachi S-5200 cryo scanning electron microscope (Hitachi).

### 2.5. Imprinting and Rebinding

For imprinting, functionalized silica particles were suspended in PBS (4 mg SP/mL) by ultrasonication and incubated with AdV5 (1.0 × 10^4^ IU/5 µL) for 30 min at 4 °C and 700 rpm. TEOS (0.45 µmol/mg SP), APTES (0.085 µmol/mg SP), and APTMS (0.11 µmol/mg SP) were added and the polymerization proceeded at 4 °C and 700 rpm for 6 h. Afterwards, the particles were washed three times with PBS. For template extraction, the particles were incubated with 0.5 M hydrochloric acid with 0.005% Triton X-100 for 30 min and washed 5 times with PBS. Non-imprinted control particles were synthesized accordingly, without the addition of template virus. For rebinding studies, 100 µL of the protein solution (1–3% BSA with 0.05% Tween 20 in PBS or 0.1–0.5% skim milk with 0.05% Tween 20 in PBS) were added to the VIPs and NIPs, directly followed by the addition of 5 µL AdV (1.0 × 10^4^ IU). After incubation for 30 min at RT on a rotating system, the suspension was centrifuged and the supernatant was analyzed via qPCR.

### 2.6. Binding Kinetics

First, 20 mg of SP were incubated with an appropriate amount of adenovirus (1.0 × 10^4^ IU/5 µL) in 5 mL PBS at 4 °C and 700 rpm. In order to determine the binding kinetics, samples were taken after different incubation times and the supernatants were analyzed via qPCR. Low binding vials were used for all experiments to reduce unspecific virus binding to the surface of the vessels.

### 2.7. AdV5 Detection in CCS

Undiluted cell culture supernatant was spiked with 5 µL AdV5 containing 1.0 × 10^4^ IU and VIPs and NIPs were incubated for 30 min at room temperature on a rotating system. The supernatant was analyzed by UV-Vis spectroscopy on a NanoDrop 2000c (ThermoFisher), as well as via sodium dodecyl sulphate–polyacrylamide gel electrophoresis (SDS-PAGE) and qPCR afterwards, to determine the amounts of proteins and virus, respectively.

### 2.8. qPCR

Prior to qPCR quantification, viral DNA was isolated using PureLink^TM^ Viral RNA/DNA mini kit (ThermoFisher) according to the manufacturer’s instructions and the measurements were performed using a Step One Plus Real-Time PCR System (Applied Biosystems, Waltham, MA, USA) with the primer and probe sequences: forward primer 5′-GTC CAT GGG CGC ACT CA-3′, reverse primer 5′-GGC GGA GTT GGC GTA GAG A-3′, probe 5′-6-FAM-ACC TGG GCC AAA AC-MGB-3′. Cycling conditions included a prior heating step to 95 °C for 10 min followed by 40 cycles at 95 °C for 15 s and detection at 60 °C for 1 min.

### 2.9. SDS-PAGE

Samples were mixed with Laemmli buffer and heated to 95 °C for 10 min. Then, 30 µL of sample mixture was applied into each well and the gel was running at 200 Volt constant for 35–37 min in TGS running buffer. The gel was stained with Coomassie blue stain overnight and washed with water.

## 3. Results and Discussion

### 3.1. Synthesis and Functionalization of Silica Core Particles

**Figure 1:**
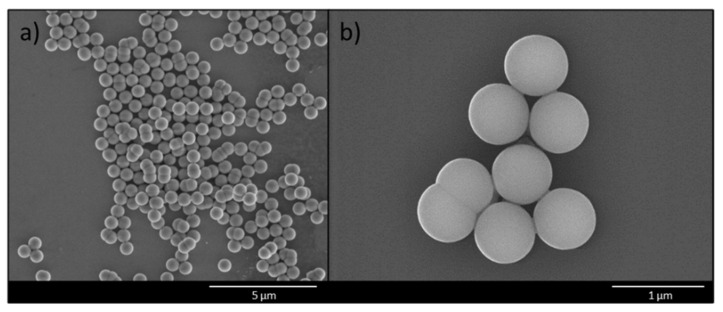
Scanning electron microscopy images of (**a**) silica core particles and (**b**) functionalized silica core particles.

Silica particles were synthesized according to the method of Stöber [24] and used as basis for the surface imprinting. These particles are characterized by a high stability and provide a high surface area for later virus binding. The surface area of the silica core particles was determined by nitrogen adsorption-desorption measurements and resulted in 10.994 ± 0.155 m^2^/g providing a suitable surface area for virus immobilization and the following surface imprinting procedure. Characterization of the core particles via SEM revealed particles with a mean diameter of around 600 nm with a uniform size distribution (Figure 1a).

Particles were functionalized in two steps to enhance the virus immobilization. Amino groups of the side chains of the viral capsid proteins can interact with aldehyde groups on the core particles forming imine bonds. This procedure facilitates the following imprinting step, since the template virus is covalently bound to the core particles. Simple washing steps with PBS could not extract the virus, confirming the strong binding of virus towards the particles. The zeta-potential represents the surface charge of the particles and, as expected, the bare silica particles possessed a negative charge from the surface hydroxyl groups (Table 1). In a first step, the functionalization with APTES was examined qualitatively by treating the particles with a ninhydrin solution. The particles showed a violet color and were then further characterized by measuring the zeta-potential. The zeta-potential changed to a positive value, which can be attributed to the positively charged amino groups. With the last functionalization step, the zeta-potential decreased again due to the introduced aldehyde groups.

In addition, successful functionalization was confirmed by XPS analysis (Table 2). For silica-APTES samples, the N 1s peak at 399 eV is visible in the spectra, which is characteristic for the amino group [25,26]. Increase of carbon concentration was monitored during the functionalization, since carbon containing groups were introduced in both steps. Additionally, amino-functionalization was confirmed by elemental analysis resulting in 1.1 ± 0.1% nitrogen content of silica-APTES particles. SEM image after the modification of the core particles with both functionalization reagents are shown in Figure 1b.

### 3.2. Binding Kinetics

**Figure 2:**
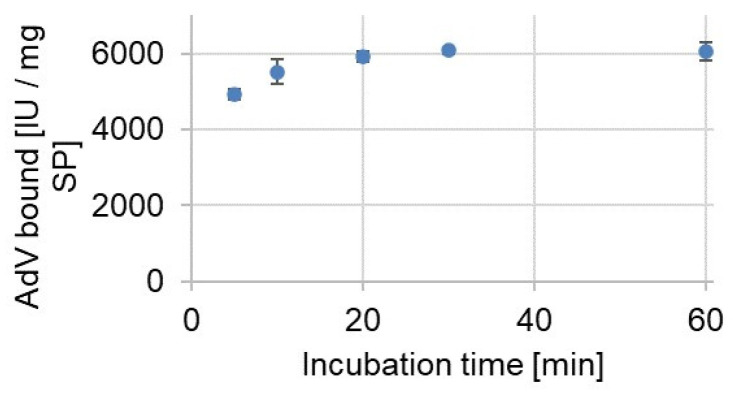
Determination of the binding kinetics of AdV5 to core particles: 20 mg functionalized silica particles were incubated with AdV5 (1.0 × 10^4^ IU/5 µL) in PBS, and samples were collected after different incubation times. After centrifugation, remaining AdV5 in the supernatant was quantified via quantitative PCR.

Functionalized silica particles were incubated with AdV5 to determine the binding kinetics. As shown in Figure 2, the virus bound to the particles and a maximum binding capacity was reached after 30 min incubation time. Due to the functionalization, the viruses can form covalent imine bonds via the amino groups of the capsid protein’s side chains with the aldehyde groups at the particle surface. Side chains of the amino acids asparagine, glutamine, lysine, and arginine can contribute to the bond formation. Imine bonds are stable at neutral pH values and the viruses could not be removed by simple washing steps with PBS, providing optimal conditions for the following imprinting procedure. With the successful virus immobilization, surface imprinting was the preferred strategy to obtain binding sites located at the surface of a solid support. Upon rebinding, better accessibility of the recognition sites can be achieved owing to the size of the target virus.

### 3.3. Imprinting and Rebinding

Imprinting was carried out with organosilane-based monomers that can bind to the silica by hydrolysis and condensation reactions forming a polymer layer around the cores. The resulting VIPs could bind high amounts of adenovirus, confirming high affinity of the virus towards the imprinted layer. After imprinting, virus extraction was verified using viral lysis buffer, which destroyed the viral capsid and the released DNA was detected via qPCR. Hence, it was confirmed that the templates were not covered by the polymer layer, since 102 ± 4% of the added virus were recovered.

**Figure 3:**
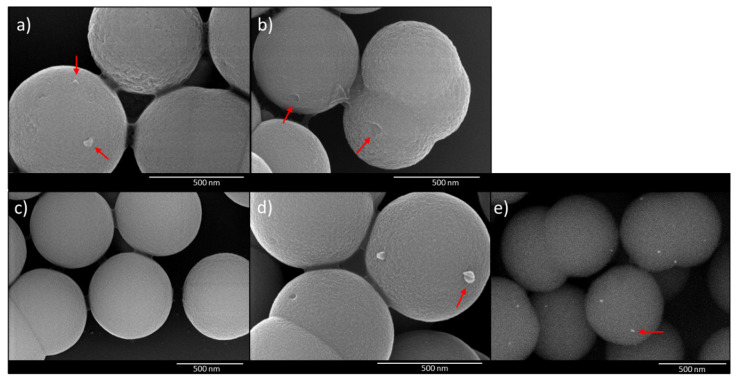
Scanning electron microscopy images of VIP before template extraction (**a**), VIP after template extraction (**b**), NIP (**c**), and VIP with rebound AdV (**d**,**e**). Samples (**a**–**d**) were coated with platinum and particles in (**e**) were treated with osmium tetroxide and coated with carbon. Exemplary arrows show potential binding sites in (**b**) and virus particles in (**a**,**d**,**e**).

The structural configuration of the template virus needs to be considered during the imprinting procedure. Therefore, PBS was used as solvent during both steps, i.e., imprinting and rebinding, since it provides a physiological environment that can stabilize the configuration of the virus capsid. Imprinted particles and VIPs with rebound adenovirus were characterized by SEM and the images are shown in Figure 3.

The polymer layer is visible at the surface of the imprinted particles. Before extraction, the template virus is located on the VIP (Figure 3a). After template extraction, the putative binding sites on the particles are marked with arrows in Figure 3b. The size of the cavities corresponds with the size of adenovirus type 5, which ranges from 70–100 nm [27,28]. Crater-like binding sites in a virus-imprinted layer were also shown by Cumbo et al. [29]. The surface of the corresponding NIPs (Figure 3c) appeared smooth and the differences between imprinted and non-imprinted particles are visible. Upon rebinding, the particles are incubated with adenoviruses, which can then bind to the created binding sites in the recognition layer (Figure 3d). However, the size and shape of the viruses can change during sample preparation for electron microscopy owing to drying and coating steps. VIPs with rebound virus were additionally treated with osmium tetroxide to verify the binding of AdV5 to the imprinted layer. Osmium tetroxide is used for sample preparation in electron microscopy to stain biological samples. The stained viruses therefore appeared as bright spots on the images resulting from the high electron density of osmium. Figure 3e shows the image of AdV-VIPs confirming the binding of the virus to the imprinted particles.

### 3.4. Rebinding in the Presence of Different Blocking Agents

For the rebinding steps, different variations of commonly used blocking agents were tested. Some proteins, including serum albumins and milk proteins, as well as non-ionic detergents, like Tween 20, are frequently used for the reduction of unspecific binding in enzyme-linked immunosorbent assays (ELISA) [30,31]. Herein, BSA and skim milk are used in combination with Tween 20 in two different concentrations each, since they are well known to reduce unspecific protein binding, and this procedure is applied to examine their ability to reduce unspecific virus binding.

The template concentration used during imprinting may affect the imprinting factors, as the number of binding sites formed may change depending on the concentrations. In addition, the structure of the virus needs to be considered, as the large viral capsid has several functional groups on the surface that could provide different interaction opportunities for unspecific binding during rebinding. Therefore, the concentration of the template was kept constant during the experiments and the influence of blocking agents on the unspecific binding of the viruses to the polymer layer was investigated.

**Figure 4:**
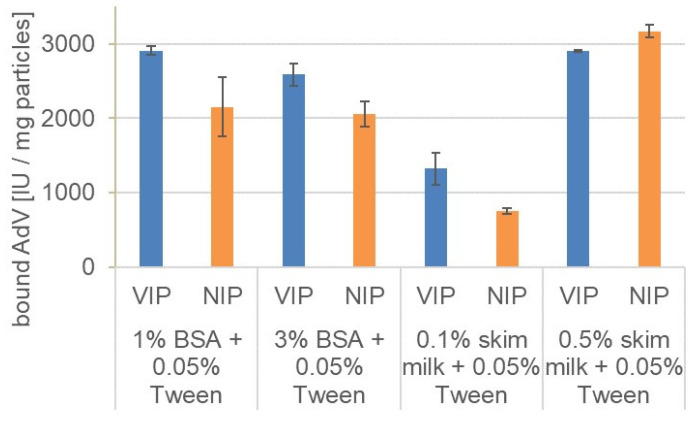
Rebinding of AdV5 to VIPs (blue) and NIPs (orange) in presence of different blocking agents. VIPS and NIPs were incubated with 100 µL protein solution (1–3% BSA with 0.05% Tween 20 in PBS or 0.1–0.5% skim milk with 0.05% Tween 20 in PBS) and AdV (1.0 × 10^4^ IU/5 µL). AdV5 remaining in the supernatant after 30 min was quantified by qPCR.

Each blocking agent solution was added to the VIPs and NIPs directly before the addition of the virus to block unspecific binding. The use of BSA and skim milk could not prevent unspecific binding completely, since significant amounts of AdV5 also bound at the NIPs. However, some differences are apparent (Figure 4).

Using BSA as blocking agent, unspecific binding was slightly reduced and the binding capacities of VIPs were in a comparable range for both BSA concentrations. Additionally, the imprinting factors (Table 3) of 1.35 with 1% BSA and 1.26 with 3% BSA were similar. Skim milk showed concentration dependent results with higher imprinting factor at lower skim milk concentrations. However, the binding capacity decreased and less virus bound to the particles at 0.1% skim milk. Use of higher concentrations (0.5% skim milk) led to higher virus binding; however, no significant differences were observed between VIPs and NIPs. Skim milk could therefore not reduce the nonspecific binding of the virus. VIPs and NIPs presented similar binding capacities towards the virus in the absence of blocking agents. Although the use of 0.1% skim milk with 0.05% Tween 20 resulted in the highest imprinting factor, the amount of bound target was reduced. Despite skim milk presented better blocking action, the use of BSA as a blocking agent resulted in higher binding capacity, which can be an advantage depending on the application.

### 3.5. Binding of AdV5 from Cell Culture Supernatant

**Figure 5:**
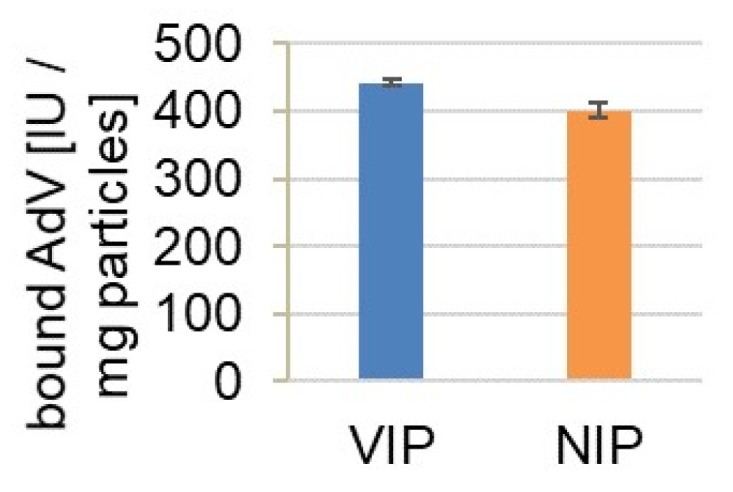
Binding of AdV from undiluted cell culture supernatant (CCS): 20 mg of VIPs (blue) and NIPs (orange) were incubated with CCS, which was spiked with AdV5 (1.0 × 10^4^ IU/5 µL) before. After 30 min incubation time, free virus in the supernatant was quantified.

Since AdV5 revealed high binding affinities for the polymer particles in PBS, its binding behavior from a complex protein mixture was examined. To simulate a virus isolation procedure virus-free cell culture supernatant was spiked with a defined amount of AdV5 and the particles were incubated with the suspension. The supernatant was analyzed by qPCR for virus quantification and by UV-Vis and SDS-PAGE for protein analysis. AdV5 showed high affinity towards the particles unaffected by the present proteins (Figure 5).

**Figure 6:**
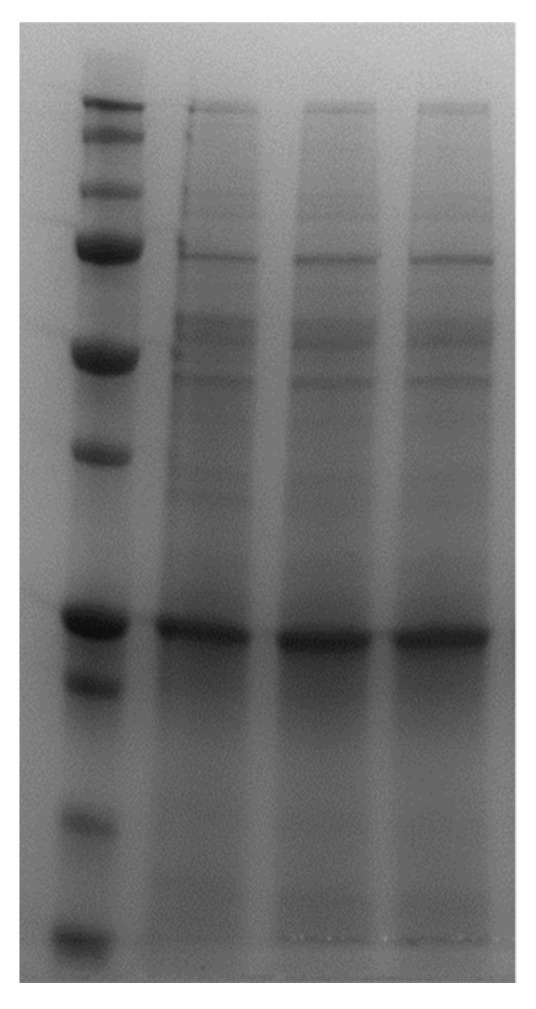
Coomassie blue stained SDS-PAGE analysis of CCS samples after incubation of VIPs and NIPs. Lane 1: molecular weight marker, lane 2: CCS, lane 3: supernatant of VIP incubated with CCS, lane 4: supernatant of NIP incubated with CCS. All samples were diluted 1:4 in PBS.

Samples were also applied for SDS-PAGE and no significant protein binding was visible since the band intensities of the initial CCS (Figure 6, lane 2) are comparable to the supernatant bands after incubation of VIPs and NIPs (lanes 3 and 4, respectively). The proteins had low binding affinity towards the polymer layer and, therefore, AdV5 could be extracted from the matrix.

**Figure 7:**
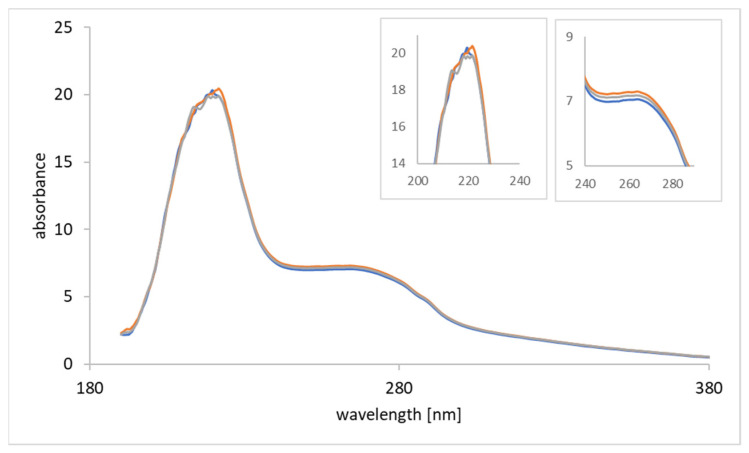
UV-Vis analysis of CCS samples. UV-Vis spectra of cell culture supernatant (blue), supernatant of VIP incubated with CCS (orange), supernatant of NIP incubated with CCS (grey). Zoom into the wavelength range 200–240 nm (**left**) and 240–280 nm (**right**).

An aliquot of the supernatant was additionally analyzed by UV-Vis (Figure 7). No significant binding of proteins and nucleic acids at both kinds of particles was obtained confirming the extraction of AdV5 from the matrix. The absorbance was similar in the different samples, i.e., the CCS, supernatant of VIP incubation and supernatant of NIP incubation. AdV5 extraction in cell culture supernatant resulted in decreased imprinting factors, compared to the binding in presence of protein blocking solutions, probably attributed to the influence of the matrix proteins, which could enhance unspecific binding. However, only negligible binding of the matrix components to the particles was obtained. Additionally, smaller compounds, like residual cellular DNA, did not bind to the VIPs, since there was no change in absorption at 260 nm. At the concentrations used in these experiments AdV5 had no influence on absorption. Surprisingly, both VIPs and NIPs showed similar binding capacities for AdV5 in CCS. The negligible binding of proteins and nucleic acids at the imprinted layer indicated potential use of NIPs for AdV5 detection and extraction in complex matrices.

## 4. Conclusions

Even though there was low preferential binding of AdV5 to the VIPs, it was shown that AdV5 could be extracted from a complex matrix with negligible unspecific protein binding to the particles. This indicated a possible application of the particles as sorbent materials for enrichment and extraction of adenovirus from different matrices such as but not limited to biological or medicinal samples. While the selectivity obtained in the presence of protein blocking agents was reduced during the binding experiments in CCS, substantial amounts of adenovirus were bound to the sorbent facilitating reliable target extraction from complex matrices. Imprinted materials are characterized by a higher stability in every respect compared to natural receptors. In addition, a high binding affinity of the target virus towards the imprinted sorbent was confirmed. While for extraction of AdV5 from CCS, NIPs also appear suitable, it is anticipated that further future optimization of the developed materials will lead to an increased selectivity.

## Figures and Tables

**Table 1:** Zeta-potentials of bare and functionalized silica particles in 1 mM KCl at 25 °C.

Particle	Zeta-Potential [mV]
silica	−49.5 ± 3.5
silica-APTES	31.8 ± 5.1
silica-APTES-glutaraldehyde	10.8 ± 4.6

**Table 2:** Atomic concentrations of bare and functionalized silica particle surfaces determined by XPS.

	Atomic Concentration [%]			
Particle	C 1s	N 1s	O 1s	Si 2p
silica	12.76	0.14	57.12	29.97
silica-APTES	16.72	0.94	52.29	30.05
silica-APTES-glutaraldehyde	22.14	1.21	49.49	27.16

**Table 3:** Imprinting factors of the rebinding studies with AdV5 in presence of different blocking solutions.

Blocking Solution	Imprinting Factor
1% BSA + 0.05% Tween 20	1.35
3% BSA + 0.05% Tween 20	1.26
0.1% skim milk + 0.05% Tween 20	1.76
0.5% skim milk + 0.05% Tween 20	0.92

## Data Availability

Data are contained within the article.
